# Soil carbon is a useful surrogate for conservation planning in developing nations

**DOI:** 10.1038/s41598-019-40741-0

**Published:** 2019-03-07

**Authors:** Pablo L. Peri, Romina G. Lasagno, Guillermo Martínez Pastur, Rachel Atkinson, Evert Thomas, Brenton Ladd

**Affiliations:** 1 Instituto Nacional de Tecnología Agropecuaria (INTA), 9400 Río Gallegos, Rio Gallegos, Argentina; 2 Universidad Nacional de la Patagonia Austral (UNPA)-CONICET, 9400 Río Gallegos, Rio Gallegos, Argentina; 3 Laboratorio de Recursos Agroforestales, Centro Austral de Investigaciones Científicas (CADIC CONICET), 9410 Ushuaia, Argentina; 4 Bioversity International, c/o CIP Avenida La Molina, 1895, La Molina, Lima, 12 Peru; 5 School of Biological, Earth and Environmental Sciences, University of New South Wales, Sydney, 2052 Australia; 6 Escuela de Agroforestería, Universidad Científica del Sur, Lima, 33 Peru

## Abstract

Defining the optimal placement of areas for biodiversity conservation in developing nations remains a significant challenge. Our best methods for spatially targeting potential locations for biodiversity conservation rely heavily on extensive georeferenced species observation data which is often incomplete or lacking in developing nations. One possible solution is the use of surrogates that enable site assessments of potential biodiversity values which use either indicator taxa or abiotic variables, or both. Among the plethora of abiotic variables, soil carbon has previously been identified as a potentially powerful predictor for threatened biodiversity, but this has not yet been confirmed with direct observational data. Here we assess the potential value of soil carbon for spatial prediction of threatened species using direct measurements as well as a wide range of GIS derived abiotic values as surrogates for threatened plant species in the PEBANPA network of permanent plots in Southern Patagonia. We find that soil carbon significantly improves the performance of a biodiversity surrogate elaborated using abiotic variables to predict the presence of threatened species. Soil carbon could thus help to prioritize sites in conservation planning. Further, the results suggest that soil carbon on its own can be a much better surrogate than other abiotic variables when prioritization of sites for conservation are calibrated on increasingly small sets of observation plots. We call for the inclusion of soil carbon data in the elaboration of surrogates used to optimize conservation investments in the developing world.

## Introduction

While achieving conservation goals requires a landscape level approach, protected areas play an important role and an optimal protected area network should contain representative examples of the ecosystems and biodiversity present in a given region. Unfortunately protected area networks rarely achieve this aim. Ecosystems and landscapes with little to no productive value are more often than not over represented^1–3^. In response to this problem, numerous solutions have been proposed to identify a minimum set of areas that are representative. Examples include the concept of the biodiversity hotspots^4^, ecoregions^5^, important bird areas^6^, the approach used by the alliance for zero extinction sites, as well as site selection based on level of threat (irreplaceability and vulnerability). These have all led to suggestions for a more systematic approach to define priorities for conservation. There has also been discussion around the need to incorporate stakeholders into the decision making process^7^, to determine the relative costs of different strategies^8^, and to consider the fact that neither biodiversity nor threats are static in space or time^9^. However, as many countries with high levels of biodiversity often lack suitable information for identifying important areas for biodiversity conservation there has been a shift toward ecosystem-based planning for the expansion of protected area networks^10,11^. Perhaps the most promising approach towards more objective conservation planning in data poor regions is the development of biodiversity surrogates which have been developed using both abiotic variables^12,13^ and indicator taxa. The incorporation of abiotic factors that describe environmental variability in particular have shown promise for surrogate elaboration^13,14^. However despite the significant progress there is a need for further development and refinement of biodiversity surrogates in conservation planning^13^.

An important variable that has previously been overlooked or missed in the elaboration of biodiversity surrogates is soil carbon. Locations with rich fertile soil in which soil carbon is abundant are an attractive target for conversion to agriculture and this has been the case since the onset of the Neolithic farming revolution^15^. As a result locations with fertile, carbon rich soils are poorly represented in existing reserve networks^2^ and the biodiversity that specializes in high-carbon soils may have highly reduced and fragmented ranges^6^. In addition, areas with carbon rich soils are likely subject to more intense anthropogenic threats related to factors such as eutrophication, invasive species, over harvesting, and land use change. We thus hypothesize that species that exist in areas where soil carbon is relatively abundant may face a larger number of more intense extinction threats than species that occur in habitats where soil carbon stocks are relatively low. Here we use observational data from the PEBANPA network of long term biodiversity plots in Southern Patagonia to assess the relationship between soil carbon and threatened plant species. This in turn allows us to test the potential value of soil carbon as a variable that could be used to improve the efficacy of surrogates used for biodiversity conservation planning.

## Methods

**Figure 1:**
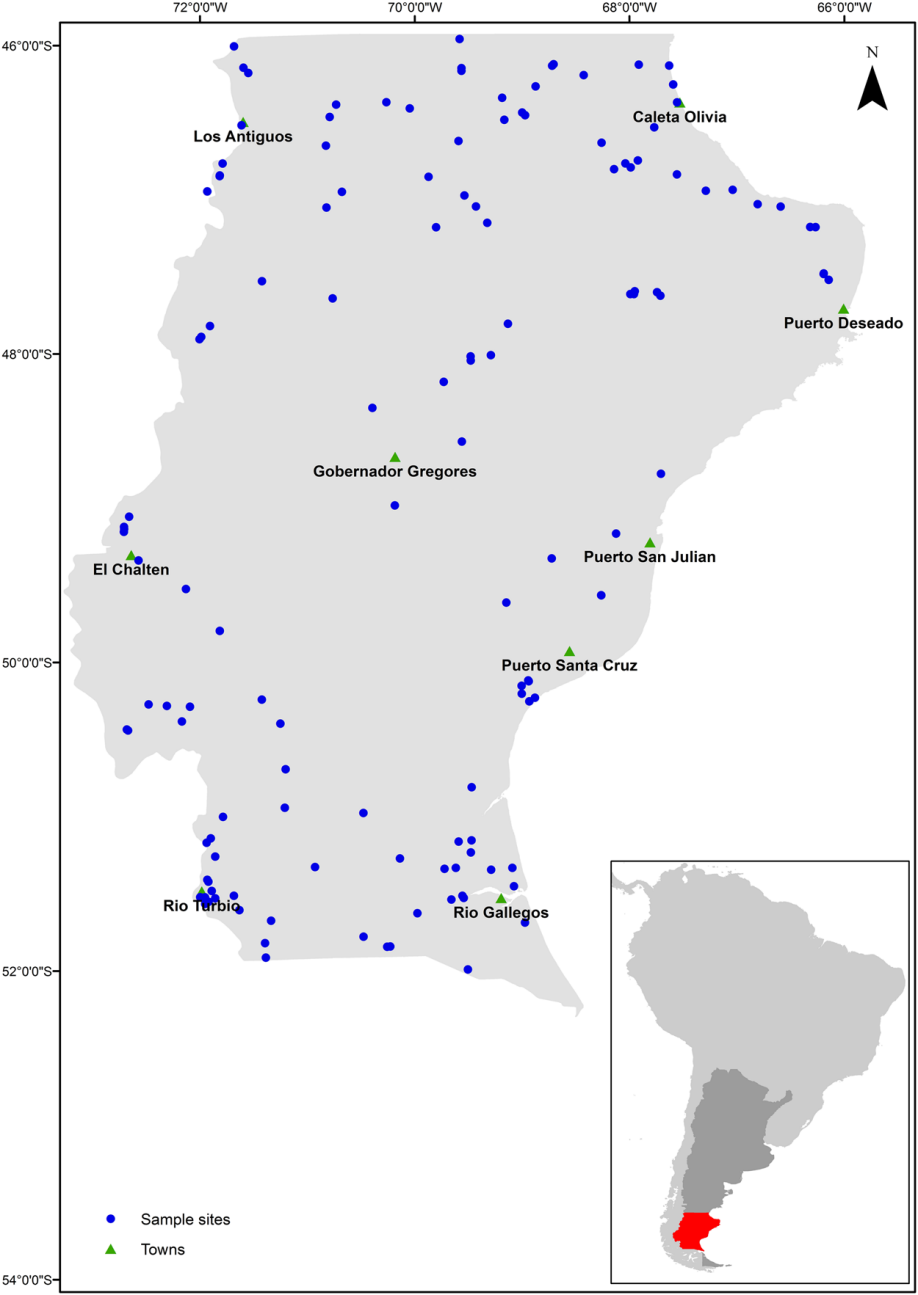
Locations of sample sites in the PEBANPA network of permanent plots in Southern Patagonia.

To assess for a possible relationship between threatened plant biodiversity and soil carbon we analyzed data from the PEBANPA network of long term biodiversity plots (Biodiversity and Ecological long-term plots in Southern Patagonia)^16^ (Fig. 1). The PEBANPA plots were established over 12 years ago and encompass native forest, grassland, shrub-land and wetlands. For each plot comprehensive botanical survey data exists as well as detailed information on climate, topography, land form, soil properties etc.^16^. To investigate possible links between soil organic carbon (SOC) stocks and threatened plant biodiversity, we extracted plant survey data for all 145 sites in the PEBANPA network.

All PEBANPA plots are permanently marked and assessed at least once during the flowering period (spring-summer) for accurate plant identification. At each sampling location, plant diversity is measured in a 20 m × 50 m quadrat (1000 m^2^). This plot size enables regional comparisons in diversity-associated factors for the broad vegetation types (e.g. grasslands, shrublands and forests). Species were classified according to origin (native, endemic, exotic), life-form (herb, graminoids, tussock grass, fern, shrub, dwarf shrub, tree), life-span (perennial, annual, biennial), and location of the plant’s growth-point (meristem) based on Raunkiaer classification system (geophyte, chamaephyte, phanerophyte, hemicryptophytes, cryptophytes, therophytes).

We used the PlanEAr website (http://www.lista-planear.org/) to identify species at risk of extinction in the PEBANPA plots. To assess the importance of different environmental variables in explaining presence or absence of these threatened species in the sample plots we used Random Forests. Given that the majority of plots contained zero or one threatened species, we used presence-absence as a response variable. The explanatory variables we used were soil C stock to 30 cm and the GIS variables described in Peri *et al*.^17^ that relate to climate, topography and land use. We reduced collinearity of variables through iterative calculations of variance inflation factors (VIF), retaining only variables with VIFs smaller than 3. To quantity variability in variable importance scores we developed 20 random forest models based on random selections of 70% of the PEBANPA plots. Importance values were based on the mean decrease in the Gini index and were standardized across runs by dividing by the value of the most important variable.

**Figure 2:**
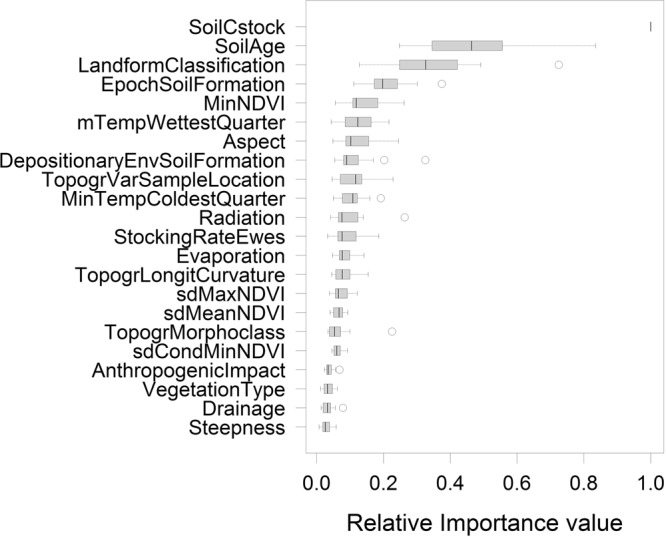
Boxplots of relative importance values of the environmental variables based on mean decrease in Gini index across twenty random runs of random forest models. Soil Age = number of years before present that the soil on site formed, Landform Classification = the landform classification of Meybeck *et al*.^23^, EpochSoilFormation = Epoch when the soil on site was formed, MinNDVI = minimum Normalized Difference Vegetation Index, mTempWettestQuarter = mean Temperature of the Wettest Quarter, DepositionayEnvSoilFormation = Depositionary Environment during Soil Formation, TopogrVarSampleLocation = Topographic Variability in a 500 m diameter spatial window around the Sample Location, mTempcoldestQuarter = mean Temperature of the Coldest Quarter. More detailed description of variable labels of lesser important is given in Peri *et al*.^29^.

To assess the usefulness of soil carbon for identifying priority sites for the conservation of threatened plant species, we assembled surrogates based on abiotic variables including and excluding soil carbon following the method proposed by Albuquerque and Beier^12^. This approach mimics the planning situation in which species data are available for only a subset q%, of the planning plots. It uses species data in each subset to determine the conservation importance of plots based on (i) an optimal conservation scenario and (ii) the use of a surrogate derived from a set of environmental variables characterizing the planning area. Under the optimal conservation scenario, the conservation scores of plots in each subset reflect their importance for finding the smallest set of geographical units that maximizes species representation. Here we used the species richness algorithm originally proposed by Rebelo and Sigfried^18^ to this end. Importance scores ranged from 0 (plot not part of the optimal conservation scenario) to 1 (plot with highest richness of unprotected species). Next, we developed random forest models^19^ to predict plot conservation importance scores in each subset from the explanatory variables shown in Fig. 2, using the scores from the optimal conservation scenario for model calibration. Model predictions for the entire planning area were then used to assess their efficiency for prioritizing conservation plots, compared to the optimal conservation scenario and the random selection of plots, through calculation of the species accumulation index (SAI)^20^ for values of q ranging from 5 to 60%. The SAI measures surrogacy value by comparing S, the number of species represented in sites selected using the surrogate (here random forest model predictions), with O, the largest number of species that can be represented in the same number of sites (optimal conservation scenario), and with R, the number of species represented in the same number of randomly selected sites. SAI was calculated as SAI = (S-R)/(O-S) whereby S represents the number of unique species averaged across 100 random forest models per q, and R the number of unique species averaged across 1000 randomly drawn subsets of plots. SAI values range from -∞ to 1: negative values indicate a worse than random result, near zero indicates random performance, and positive SAI values (times 100) indicate percent efficiency. For further methodological details please refer to Albuquerque and Beier^12^. All analyses were performed using the *randomForest* package^21^ for R^22^, using default settings.

## Results

**Figure 3:**
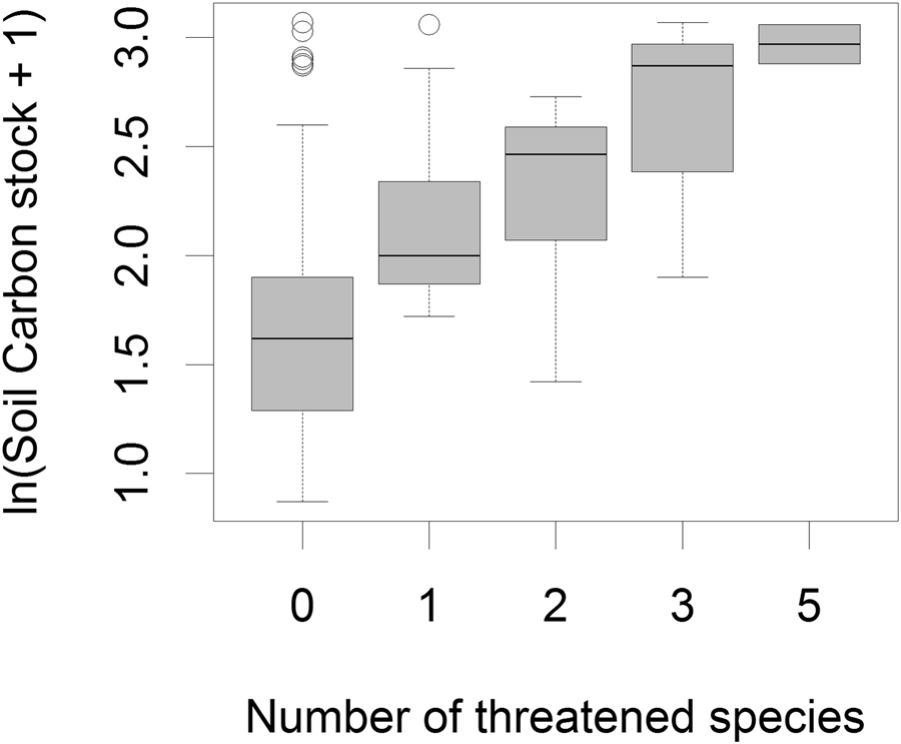
Boxplots of soil carbon values for plots with different numbers of threatened species in the PEBANPA network. Only plots with zero threatened species had significantly lower soil carbon than plots with higher numbers of threatened species p < 0.01, Tukey post-hoc test for ANOVA).

**Figure 4:**
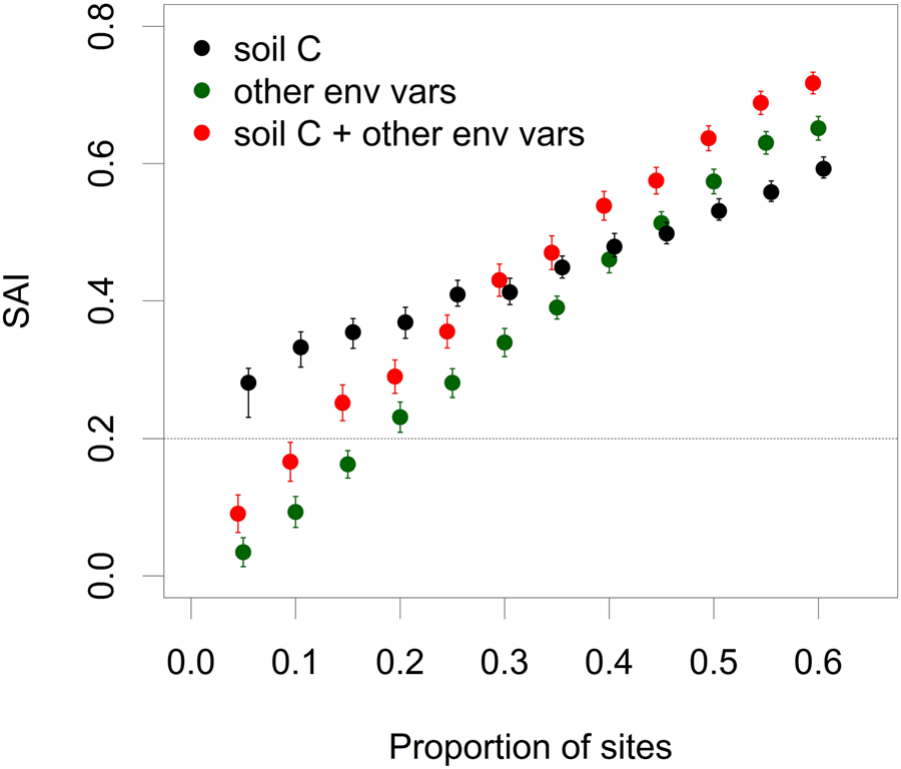
The efficiency of (i) a biodiversity surrogates based on soil carbon alone, (ii) a surrogate elaborated with environmental variables, and (iii) a surrogate elaborated with environmental variables and soil carbon for identifying priority sites for the conservation of threatened species in the PEBANPA network of permanent plots, expressed as the Species Accumulation Index (SAI). The error bars represent the 95% confidence intervals across 100 SAI values, each corresponding to a random forests model developed using the percentage of sites q indicated on the x-axis. The horizontal line at SAI = 0.2 represents the threshold above which a surrogate is considered good or reliable^12^.

In the PEBANPA network, 470 plant species from 216 genera and 68 families were recorded in the quadrats surveyed. Across the entire permanent plot network there were 37 species belonging to 20 genera and 13 families that are listed as threatened on the PlanEAr website. There was a strong trend between the number of threatened species found in plots and soil carbon (Fig. 3). The random forest models accurately predicted the presence or absence of threatened plant species in approximately 70% of the plots (70.0 ± 3.8% and 71.8 ± 6.0% for training and testing data, respectively). Soil carbon consistently featured as the most important predictor variable across the twenty random forest models and was more than twice as important than the second and third-most important variables, i.e. soil age, and the landform classification of Meybeck *et al*.^23^ (Fig. 2 and S1). SAI scores obtained showed that the inclusion of soil carbon significantly increased the efficiency of a surrogate based on abiotic variables to identify sites with the highest conservation priority (Fig. 4; red dots 7 ± 1% higher SAI scores than green dots). Remarkably, soil carbon alone was a better surrogate at q < 40% than surrogates that included all other environmental variables (Fig. 4; black dots 6–25% higher SAI scores than green dots).

## Discussion

The inclusion of abiotic factors in biodiversity surrogates appears to be a promising approach towards more objective conservation planning in data poor regions^13,14^. Here we show for the first time that including soil carbon significantly improves the performance of a biodiversity surrogate to predict the presence of threatened vascular plant species in Patagonia, adding useful knowledge that will enable further development and refinement of biodiversity surrogates used in conservation planning^13^.

We conclude that over the diverse range of ecosystems found in Southern Patagonia any effort to protect ecosystems with relatively high soil carbon stocks will also benefit threatened plant species. Prioritizing sites with relatively high soil C stocks in the conservation planning process would also help to ensure that the myriad, and poorly documented ecosystem services that flow from soil and soil carbon^24^ are also protected^6^.

While the results show a clear correlation between soil carbon and threatened biodiversity, this relationship may be coincidental rather than causal. Low lying parts of the landscape tend to accumulate organic carbon in soil and sediment due to erosional processes, slowed decomposition (anoxia) and reduced burning^25–27^. The relative abundance of water and rich fertile soil with abundant soil carbon make these same areas attractive for agriculture^15^, resulting in extensive land use change. As such, biodiversity that is specialized to these areas may become threatened due to a loss of suitable habitat, over- harvesting of wildlife, eutrophication of water bodies due to high fertilizer use and impacts of invasive species, common in highly modified landscapes, among others. Additionally, because of conflicting land uses these areas tend to be poorly represented in reserve networks^2^. Thus, species that exist in low lying areas rich in soil carbon may face a larger number of more intense extinction threats than species that occur in dry upland habitats where soil carbon stocks are generally lower.

Consistent with previous studies that have used abiotic variables to elaborate biodiversity surrogates^12^, the SAI values for the surrogate based on environmental variables was >0.20 at q ≥ 20% for the PEBANPA network. This implies that the surrogate is 20% more effective in identifying priority sites for conservation than randomly selected sites when the surrogate model is elaborated using data from 20% of the sites, indicating that the addition of soil carbon to the environmental variables increases the surrogate’s power. It is also striking is that a surrogate based only on soil carbon had an SAI value > 0.25 for q ≥ 5%, indicating that soil carbon alone is a powerful predictor for identifying sites of the highest conservation value.

However, it should be noted that while the SAI curve implies that soil carbon initially outcompetes all other variables as a standalone surrogate, it performs less well when more sites are added to the network. This is what we would expect, as it implies that although threatened species may be concentrated on sites with high soil carbon, these sites are relatively homogeneous and a few sites will be able to represent the biodiversity adapted to these areas. To add additional biodiversity to a network design will thus require inclusion of other types of area. Furthermore, relying only on soil carbon could lead to the selection of sites that are adjacent to one another; for example, if all remaining high-soil carbon sites are close together. However, as carbon-rich soils are often associated with water courses, the inclusion of these areas could improve ecological connectivity at a landscape level, increasingly important as the climate changes. Thus spatial optimization methods should always be used, even when surrogates appear to have a high degree of efficacy.

Thanks to recent advances in our ability to map soil carbon using freely available geographic datasets^28,29^ obtaining soil carbon estimates is relatively straightforward. While it is obvious that factors other than soil carbon also contribute to species extinction risk, the results presented here demonstrate that soil carbon could be a useful additional variable for the elaboration of biodiversity surrogates that aim to identify networks of sites critical for the conservation of threatened species.

## Supplementary information

Figure S1
