# Supplementary material for: Soil carbon is a useful surrogate for conservation planning in developing nations
Source: Sci Rep. 2019 Mar 7;9:3905. doi: 10.1038/s41598-019-40741-0 (PMC6405948; doi:10.1038/s41598-019-40741-0)
Supplement: Supplementary file 1 — Figure S1 [file 41598_2019_40741_MOESM1_ESM.pdf]

Supplementary Material to:

Soil carbon is a useful surrogate for conservation planning in developing nations

Pablo L. Peri [1,2,] , Romina G. Lasagno[1], Guillermo Martínez Pastur[3], Rachel Atkinson[4], Evert Thomas[4], and Brenton Ladd [5,6]*.

[1]Instituto Nacional de Tecnología Agropecuaria (INTA); 9400 Río Gallegos, Argentina

[2]Universidad Nacional de la Patagonia Austral (UNPA)-CONICET, 9400 Río Gallegos, Argentina

[3]Laboratorio de Recursos Agroforestales, Centro Austral de Investigaciones Científicas (CADIC CONICET); 9410 Ushuaia, Argentina

[4]Bioversity International, c/o CIP Avenida La Molina 1895, La Molina, Lima 12 Peru.

[5]School of Biological, Earth and Environmental Sciences, University of New South Wales, Sydney 2052, Australia

[6]Escuela de Agroforestería, Universidad Científica del Sur; Lima 33, Perú

* Author to whom correspondence should be addressed (bladd@cientifica.edu.pe).

Figure S1. Partial dependence plots for random forest model based on all PEBANPA plot data with presence/absence of threatened species as response variable. Plots show marginal effects of each variable on the probability of finding at least one threatened species.

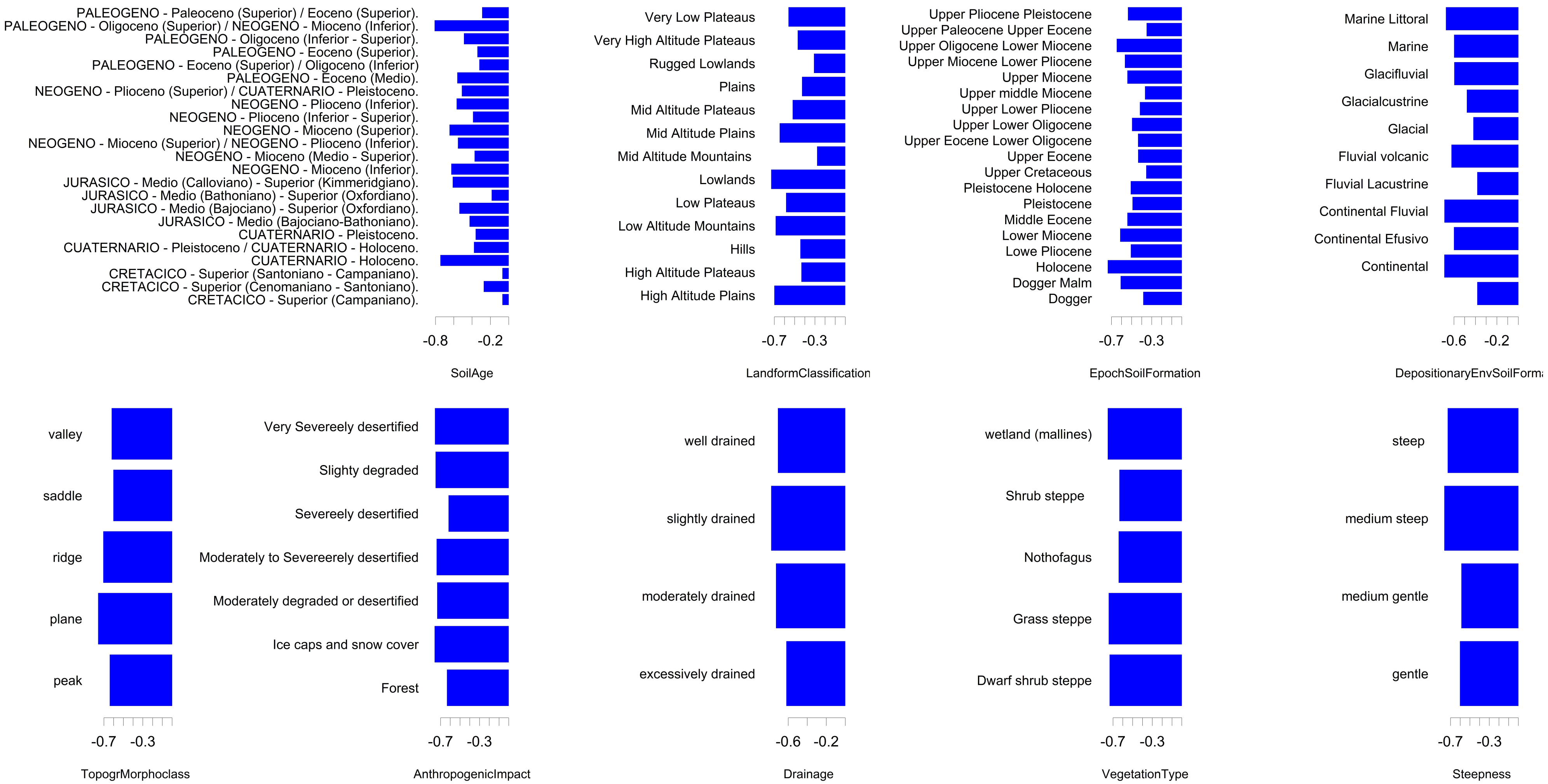
PALEOGENO - Paleoceno (Superior) / Eoceno (Superior).
PALEOGENO - Oligoceno (Superior) / NEOGENO - Mioceno (Inferior).
PALEOGENO - Oligoceno (Inferior - Superior).
PALEOGENO - Eoceno (Superior).
PALEOGENO - Eoceno (Superior) / Oligoceno (Inferior)
PALEOGENO - Eoceno (Medio).
NEOGENO - Plioceno (Superior) / CUATERNARIO - Pleistoceno.
NEOGENO - Plioceno (Inferior).
NEOGENO - Plioceno (Inferior - Superior).
NEOGENO - Mioceno (Superior).
NEOGENO - Mioceno (Superior) / NEOGENO - Plioceno (Inferior).
NEOGENO - Mioceno (Medio - Superior).
NEOGENO - Mioceno (Inferior).
JURASICO - Medio (Calloviano) - Superior (Kimmeridgiano).
JURASICO - Medio (Bathoniano) - Superior (Oxfordiano).
JURASICO - Medio (Bajociano) - Superior (Oxfordiano).
JURASICO - Medio (Bajociano-Bathoniano).
CUATERNARIO - Pleistoceno.
CUATERNARIO - Pleistoceno / CUATERNARIO - Holoceno.
CUATERNARIO - Holoceno.
CRETACICO - Superior (Santoniano - Campaniano).
CRETACICO - Superior (Cenomaniano - Santoniano).
CRETACICO - Superior (Campaniano).
-0.8
-0.2
SoilAge
Very Low Plateaus
Very High Altitude Plateaus
Rugged Lowlands
Plains
Mid Altitude Plateaus
Mid Altitude Plains
Mid Altitude Mountains
Lowlands
Low Plateaus
Low Altitude Mountains
Hills
High Altitude Plateaus
High Altitude Plains
-0.7
-0.3
LandformClassification
Upper Pliocene Pleistocene
Upper Paleocene Upper Eocene
Upper Oligocene Lower Miocene
Upper Miocene Lower Pliocene
Upper Miocene
Upper middle Miocene
Upper Lower Pliocene
Upper Lower Oligocene
Upper Eocene Lower Oligocene
Upper Eocene
Upper Cretaceous
Pleistocene Holocene
Pleistocene
Middle Eocene
Lower Miocene
Lowe Pliocene
Holocene
Dogger Malm
Dogger
-0.7
-0.3
EpochSoilFormation
Marine Littoral
Marine
Glacifluvial
Glacialcustrine
Glacial
Fluvial volcanic
Fluvial Lacustrine
Continental Fluvial
Continental Efusivo
Continental
-0.6
-0.2
DepositionaryEnvSoilForma
valley
saddle
ridge
plane
peak
-0.7
-0.3
TopogrMorphoclass
Very Severeely desertified
Slighty degraded
Severeely desertified
Moderately to Severeerely desertified
Moderately degraded or desertified
Ice caps and snow cover
Forest
-0.7
-0.3
AnthropogenicImpact
well drained
slightly drained
moderately drained
excessively drained
-0.6
-0.2
Drainage
wetland (mallines)
Shrub steppe
Nothofagus
Grass steppe
Dwarf shrub steppe
-0.7
-0.3
VegetationType
steep
medium steep
medium gentle
gentle
-0.7
-0.3
Steepness

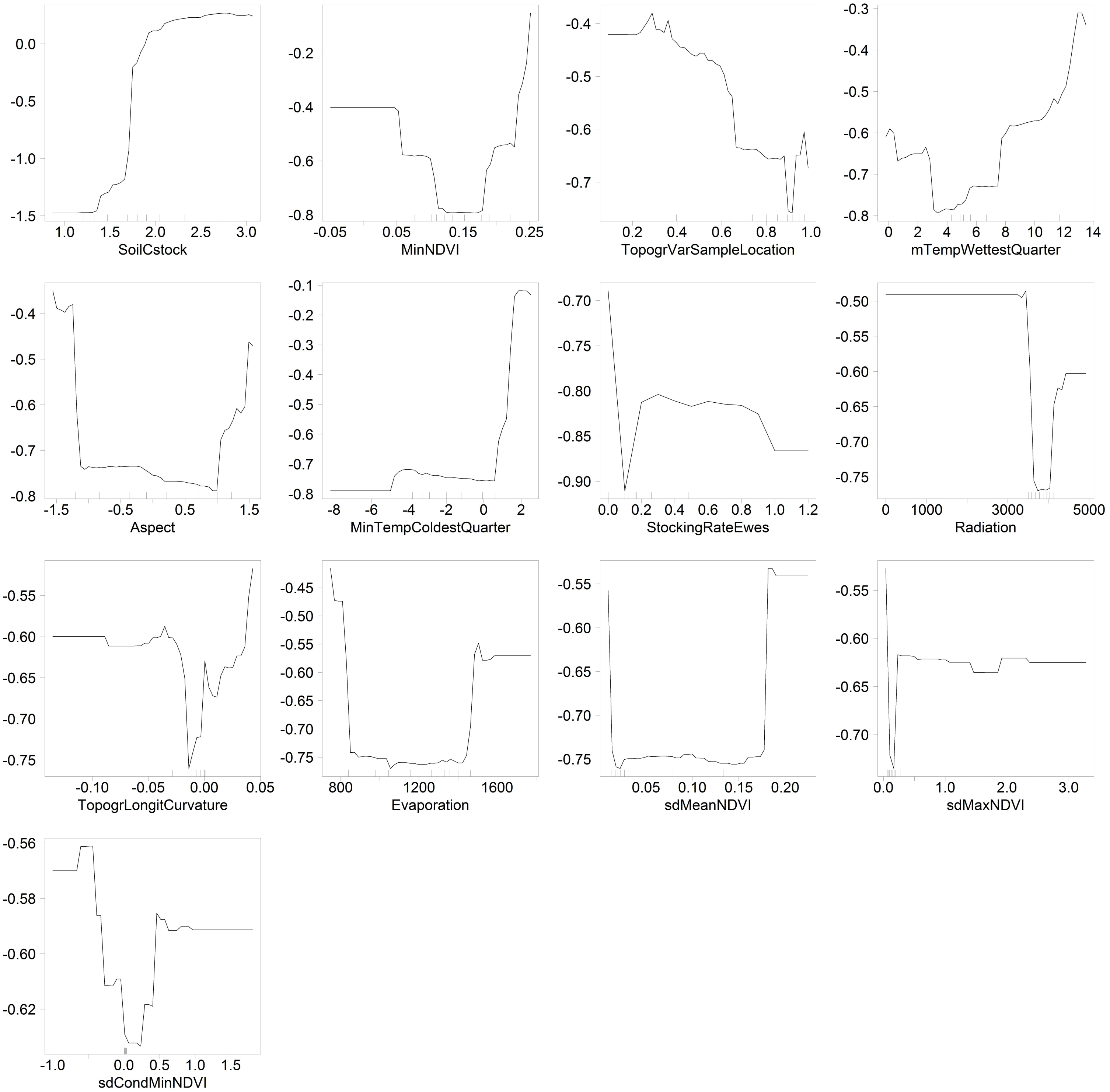
SoilCstock
MinNDVI
TopogrVarSampleLocation
mTempWettestQuarter
Aspect
MinTempColdestQuarter
StockingRateEwes
Radiation
TopogrLongitCurvature
Evaporation
sdMeanNDVI
sdMaxNDVI
sdCondMinNDVI
